# Same Genes, Different Wings: Wing Shape Divergence Among Castes in a Social Wasp

**DOI:** 10.1002/ece3.74478

**Published:** 2026-10-18

**Authors:** Kayla A. Murray, Sarah E. Orr, Michael A. D. Goodisman, Emma L. Gosse, Rachel Hill, Jennifer L. Kovacs

**Affiliations:** ^1^ School of Biological Sciences Georgia Institute of Technology Atlanta Georgia USA; ^2^ Department of Biology The University of Tampa Tampa Florida USA; ^3^ Department of Biology Agnes Scott College Decatur Georgia USA

**Keywords:** caste differentiation, genetic constraint, geometric morphometrics, polyandry, quantitative genetics, social insects

## Abstract

Wings play a central role in shaping dispersal, behavior, and ecological specialization of insects. In social insects, distinct castes with specialized morphologies arise from a shared genome, raising the question of how divergence of traits, such as wings, occurs despite genetic constraints. We investigated genetic constraints and sources of variation in wing shape in the Eastern yellowjacket wasp ( *Vespula maculifrons* ), combining geometric morphometrics and quantitative genetic analyses across queens, workers, and males. Landmark‐based Procrustes analyses revealed strong divergence in mean wing shape among castes. Males showed greater wing‐shape variation than queens and workers, consistent with weaker stabilizing selection. Allometric analyses revealed that individual‐level size‐shape relationships are weak across all castes, with no caste showing consistent significant allometry across colonies, indicating that size‐related developmental programs do not strongly couple wing shape to body size. Wing shape also varied significantly among colonies, revealing a substantial colony‐level component of wing‐shape variation that may reflect shared environmental, genetic, and socially mediated developmental influences. Additionally, we found that queens and workers from different patrilineal subfamilies differed in wing shape, demonstrating substantial heritable contributions. Our analyses also revealed limited genetic correlations between worker and queen wing shape components, suggesting that shared genetic architecture would not strongly constrain independent caste evolution of wing morphology. Together, these results show that variation in wing shape in *V. maculifrons* is shaped by a balance of genetic diversity generated by polyandry, colony‐level environmental variation, including environmental and genetic influences, and caste‐specific selective pressures associated with division of labor. Thus, caste‐specialized wing morphology can evolve in insect societies despite a shared genome and genetic constraints.

## Graphical abstract

We use a social wasp ( *Vespula maculifrons* ) as a model to address a general question: how does a single genome produce multiple, functionally distinct wing designs across castes? Our research ultimately identifies genetic effects influencing wing shape within and between castes, and provides insight into the evolution of flight and caste differences in insect.

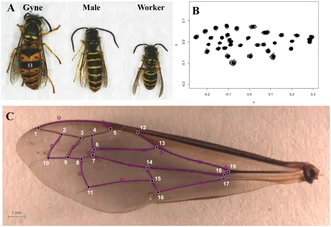

## 1 Introduction

Flight represents one of the most remarkable evolutionary innovations in insects (Kukalovapeck 1983; Misof et al. 2014). Thus, considerable research has focused on understanding the structure and mechanics of insect wings and flight mechanisms (Wootton 1992; Dudley 2000; Sane 2003; Wang 2005). Wing morphology, which includes size, shape, and venation patterns, varies widely across insect taxa and strongly influences aerodynamic performance and flight behavior (Dudley 2000; Combes and Daniel 2003; Salcedo et al. 2019). Insect wings are complex structures and vary considerably in structure and function between insect orders (Wootton 1992; Combes and Daniel 2003; Young et al. 2009; Nel et al. 2012; Hoffmann et al. 2018; Salcedo et al. 2019). Because flight is closely linked to survival and reproduction, wings provide a valuable system for understanding how ecological pressures shape morphological diversity.

Social insects present a particularly interesting context for examining variation in wing structure and function. Social insect colonies contain distinct castes that differ in physiology, behavior, and morphology (Wilson 1971, 2005; Hölldobler and Wilson 1990). These caste differences reflect strong selection associated with the division of labor. Hymenopteran social insects, for example, show great variation in wing structure among castes that reflect their different functions. For example, ants provide striking examples of extreme caste‐based wing divergence (Bourguignon et al. 2016; Peeters et al. 2020); worker ants lack wings entirely and only reproductive queens and males retain flight. Notably, however, ant queens and males may fly only one day in their entire life. In contrast, all castes of social wasps and social bees retain wings, although the castes use flight in different ways. Workers rely heavily on flight for foraging and nest defense; queens and males may fly only during mating season or colony founding (Wilson 1971; Hölldobler and Wilson 1990). These differences in flight demands suggest that wing morphology may evolve in a caste‐specific manner and that genetic correlations may strongly shape flight behavior and wing development in social insects.

Wing structure and function are likely to be influenced by environmental and genetic effects. Environmental variation can affect developmental processes and lead to variation in wing shape or structure. For example, many insects show wing polyphenisms whereby development of wings is dependent on environmental conditions (Abouheif and Wray 2002; Rajakumar et al. 2012; Ogawa and Miura 2014; Lin et al. 2018; McDonald et al. 2018; Koch et al. 2021; Du et al. 2022; Qiu et al. 2022; Li et al. 2024). Further, wing variation is also likely to be influenced by genetic variation (van't Hof et al. 2016; Iijima et al. 2019; Foster et al. 2021; Pelletier et al. 2023). Some social insect colonies are headed by multiply mated queens (polyandry) (Arnqvist and Nilsson 2000; Crozier and Fjerdingstad 2001; Hughes et al. 2008; Snook 2014; Taylor et al. 2014; Baer 2016; Saga et al. 2020). Polyandry increases intracolonial genetic diversity and, therefore, may contribute to wing variation within colonies. Shared genetic influences on wing phenotype may be particularly important to understanding phenotypic evolution of species that display distinct phenotypic classes, such as castes in social insect species (Lande 1980; Bourke 2023; Agren et al. 2024). The shared genetic architecture may lead to genetic constraint and prevent the different castes from evolving their optimum traits. Such constraint may be particularly important in the evolution of wings in social insects, where wing function may vary substantially between the castes or sexes (Hall et al. 2013; Pennell et al. 2018).

The goal of this study is to gain a better understanding of the environmental and genetic factors that affect wing shape in insect societies. We used geometric morphometric approaches to investigate wing morphology in queens, workers, and males from natural colonies of the social wasp *Vespula maculifrons* . The Eastern yellowjacket, *Vespula maculifrons* , is a common social wasp native to North America that serves as an important arthropod predator in both natural and disturbed environments. Colonies are founded by a single queen in spring and by the end of the reproductive season can contain thousands of workers and hundreds of new queens (gynes) and males (MacDonald and Matthews 1981). Queens typically mate with an average of six males, generating multiple patrilines within each nest (Ross 1986; Goodisman et al. 2007a, 2007b; Kovacs et al. 2008; Loope et al. 2014; Saga et al. 2020; Orr, Hedrick, Murray, Pasupuleti, and Goodisman 2024). This polyandrous mating system makes *V. maculifrons* a strong system for analyzing how genotype contributes to variation in morphological traits within and among castes.

Here, we combine genotypic data with landmark‐based geometric morphometrics of forewing venation to test how caste, colony‐level variation, and intracolonial genetic structure shape wing morphology. Specifically, we ask (1) whether queens, workers, and males differ in mean wing shape, consistent with their divergent roles and life histories; (2) whether patriline identity explains within‐caste variation in wing shape, indicating heritable genetic effects on wing morphology; and (3) whether genetic effects on wing shape are shared across female castes, which would reveal potential constraints on independent worker and queen evolution. We interpret caste differences in wing shape in light of well‐documented differences in caste roles and flight behavior in social wasps (Richter 2000; Martinez et al. 2021; Mattiacci et al. 2022). By integrating geometric morphometrics with known patriline structure in a polyandrous social insect, our study reveals how selection and genetic architecture jointly shape a key functional trait and illuminates the extent to which caste specialization can proceed despite a shared genome.

## 2 Methods

### 2.1 Study System and Sampling

Ten colonies of *V. maculifrons* were collected from Atlanta, Georgia in November of 2021 and 2022. The timing of collection was chosen to ensure an ample presence of gynes, workers, and males (Figure 1A) within each colony based on the traditional reproductive cycle of *V. maculifrons* . The colonies were transported to the laboratory in ventilated plastic containers, then euthanized and stored in a −20°C freezer. A total of 40–50 gynes, 100 workers, and 10 males were sorted from each colony and placed in sterile microcentrifuge tubes for subsequent analysis (Orr, Hedrick, Murray, Pasupuleti, and Goodisman 2024; Orr, Hedrick, Murray, Pasupuleti, Kovacs, and Goodisman 2024).

**FIGURE 1:**
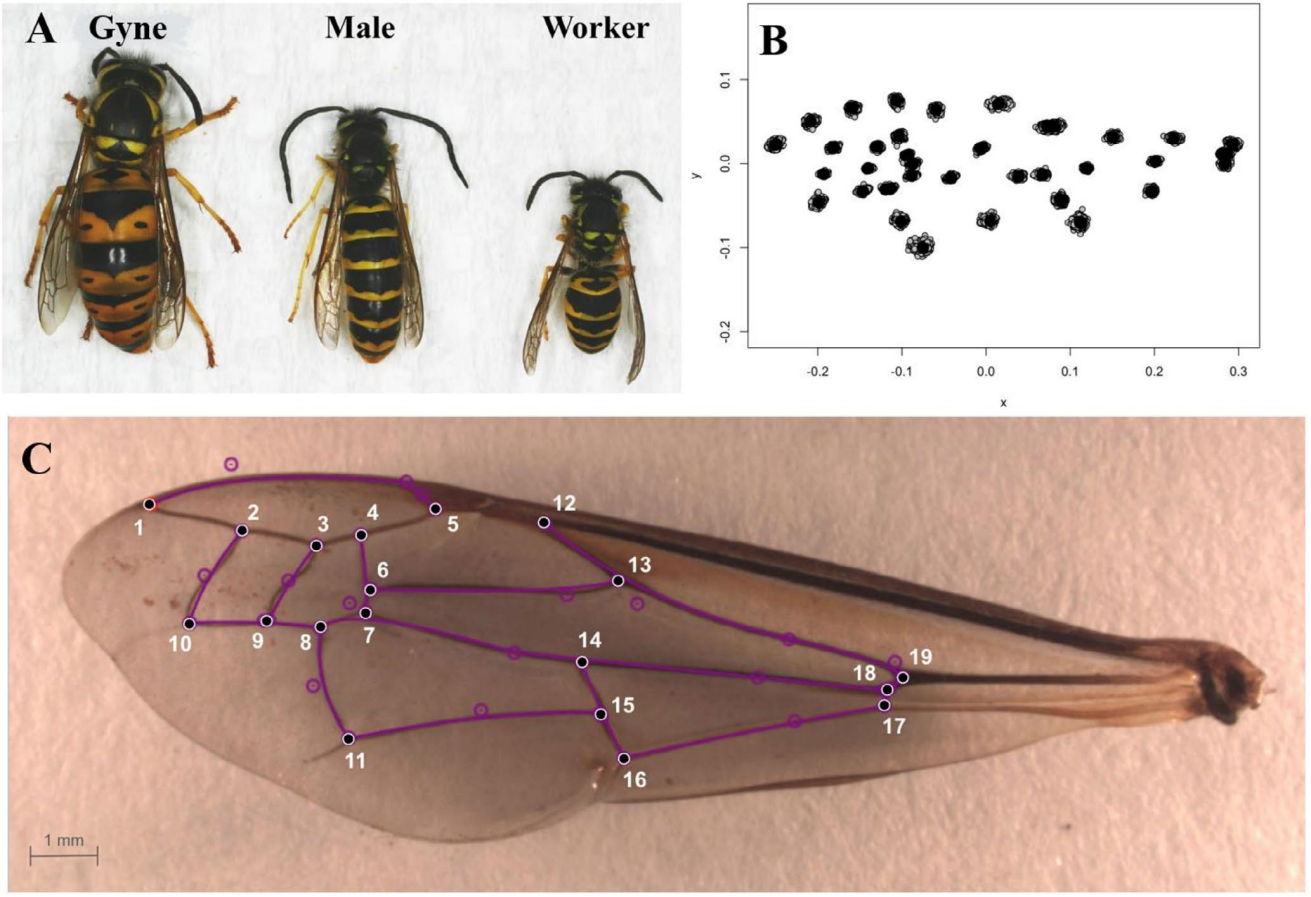
(A) Representative images of gyne, male, and worker *V. maculifrons* wasps demonstrating the phenotypic diversity among castes. (B) Generalized Procrustes Alignment of variable wing landmarks across all samples, which includes 965 individuals from 10 colonies. (C) Representative wing image with each of 19 primary landmarks and 12 landmark curves labeled as used in geometric data analysis.

### 2.2 Wing Imaging and Landmarking

The right forewing of each wasp was carefully removed from the thorax and secured in a cardboard coin holder for imaging. Wings were imaged using an Olympus SZX16 dissection microscope in conjunction with Teledyne Lumenera Infinity5 camera software. Wing images were measured using the *StereoMorph* (v1.6.7) package in R (v4.5.2). A total of 19 anatomical landmarks (Figure 1B,C), primarily defined by vein intersections and other venation features, and 12 curves tracing corresponding wing regions were manually placed on each forewing by a single researcher. These vein‐based landmarks and curves were selected based on their consistency across *Vespula* castes, ensuring comparability between individuals (Perrard et al. 2016). Additionally, they permit the quantification of overall wing shape using geometric morphometrics. Any damaged wings that obscured any of the landmarks or curves were excluded from the dataset due to the incompatibility with morphometric analysis. This process resulted in a final dataset of 965 measured wings, with approximately 100 individuals per colony (Table S1).

### 2.3 Genotyping and Patriline Assignment

DNA was extracted from a posterior leg from each individual gyne and worker. Individuals were genotyped at six highly variable microsatellite markers, following methods developed as part of a broader study of genetic and morphological variation in *V. maculifrons* (Orr, Hedrick, Murray, Pasupuleti, and Goodisman 2024; Orr, Hedrick, Murray, Pasupuleti, Kovacs, and Goodisman 2024). Microsatellite loci were amplified in duplex PCR reactions; fragment sizes were determined by Eton Biosciences for fragment analysis, and allele sizes were scored manually using ThermoFisher Connect Microsatellite Analysis software.

Microsatellite loci were used to infer relatedness and paternal‐family membership. They were not assumed to be causal markers for wing shape. Because *V. maculifrons* colonies are headed by a single polyandrous queen and females are diploid whereas males are haploid, daughters within a colony can be grouped into paternal families based on compatible multilocus genotype distributions. Individuals assigned to the same patriline share an inferred sire and are treated as full‐sib subfamilies. Individuals assigned to different partilines within the same colony share a dam but have different inferred sires and are maternal half‐sibs (Orr, Hedrick, Murray, Pasupuleti, and Goodisman 2024; Orr, Hedrick, Murray, Pasupuleti, Kovacs, and Goodisman 2024). The six‐locus microsatellite panel provided high power to distinguish sires in these colonies, with a combined non‐detection error of 0.000249 (Orr, Hedrick, Murray, Pasupuleti, and Goodisman 2024).

### 2.4 Morphometric Analyses

All geometric morphometric and statistical analyses were performed in R using the *geomorph* (v4.0.9) and *RRPP* (v2.1.2) packages (Adams and Otárola‐Castillo 2013). Landmark configurations were first aligned using the gpagen function to perform a Generalized Procrustes Alignment (GPA), which removes non‐shape variation such as differences in position, orientation, and scale among specimens (Figure 1B). Although GPA standardizes shapes to a common scale, we retained each individual's centroid size as a separate scalar measure of wing size for subsequent allometric analyses. A Principal Component Analysis (PCA) was conducted using the gm.prcomp function to summarize shape variation across all individuals. The resulting PCA plots were visualized in base R, using customized color schemes and subset selections to highlight key group differences such as caste, colony, or patriline (Figure 2A, Figure S1).

**FIGURE 2:**
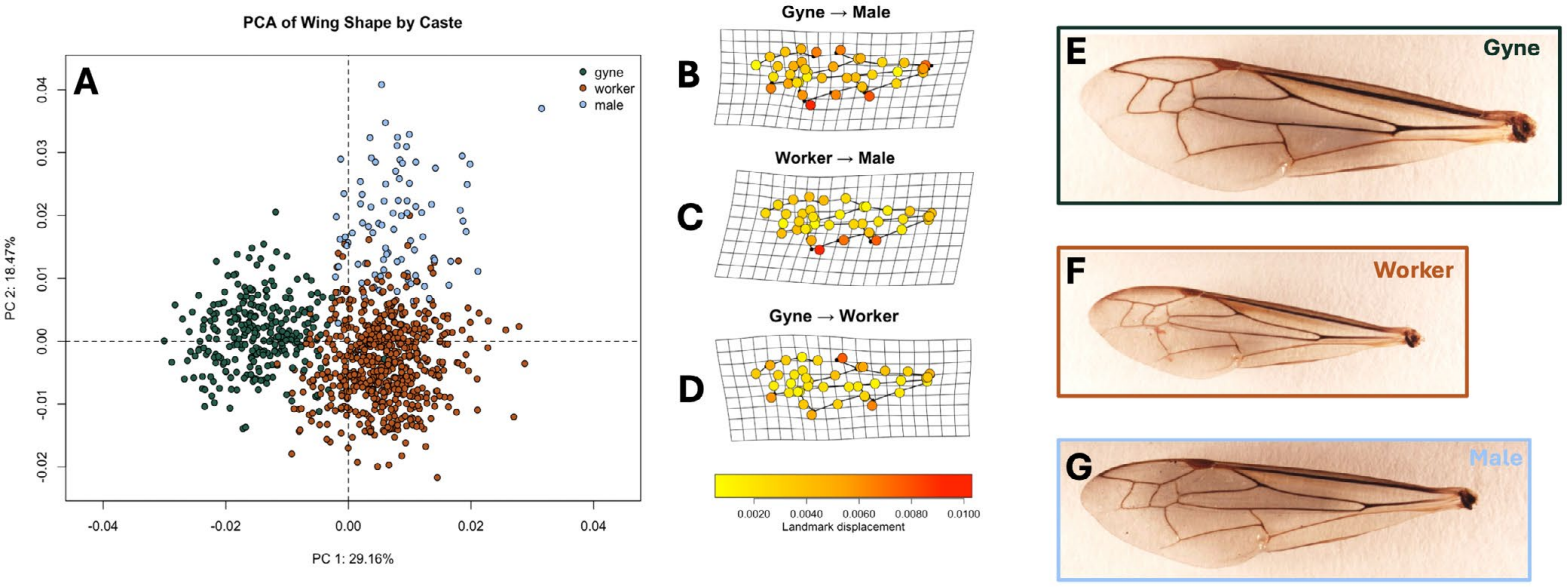
Caste differences in wing shape. (A) Principal component analysis (PCA) of Procrustes‐aligned wing shapes showing clear separation among gynes, workers, and males across all colonies. (B–D). Deformation grids with heat maps illustrating how the average wing of one caste would have to deform to match the average wing of another caste. Representative wings for gynes (E), workers (F), and males (G) shown to scale to visualize overall caste differences in wing size and shape.

Wing shape differences between castes, colonies, and patrilines were statistically evaluated using the procD.lm function to run Procrustes ANOVAs with 1000 permutations (Table 1). This test quantifies whether group means differ significantly in multivariate shape space. To illustrate shape change along specific PCA axes or between groups, deformation grids were generated using the plotRefToTarget function (Figure 2, Figures S2 and S3). Mean configurations were first calculated with mshape, then compared across castes or colonies to visualize local expansions or contractions in wing structure. We use “wing shape” to refer to the multivariate geometric configuration of our chosen 19 predominantly venation‐based landmarks and curves after Procrustes alignment. However, the term does not refer to discrete variation in vein presence, branching, or vein identity.

**TABLE 1:** Procrustes ANOVA of wing shape variation across hierarchical caste, colony, and patriline factors.

Factor	df	SS	*R* ^2^	*F*	*Z*	*p*
**A. All castes**						
Caste	2	0.12895	0.31082	291.96	12.12	0.001***
Colony (Caste)	27	0.07944	0.19149	13.32	15.40	0.001***
Residuals	935	0.20647	0.49769	—	—	—
**B. Gynes**						
Colony	9	0.02366	0.3149	18.43	18.97	< 0.001***
Patriline (Colony)	21	0.01668	0.2220	5.57	11.94	< 0.001***
Residuals	244	0.03480	0.4632	—	—	—
**C. Workers**						
Colony	9	0.03965	0.2276	24.02	16.57	< 0.001***
Patriline (Colony)	21	0.03041	0.1745	7.90	17.31	< 0.001***
Residuals	568	0.10417	0.5979	—	—	—

*Note:* Results show effects of caste, colony nested within caste, and patriline nested within colony on Procrustes‐aligned wing shape. Part A includes all three castes and partitions variance among caste, colony within caste, and residual components. Parts B and C present separate analyses for gynes and workers, respectively, testing patriline within colony effects on wing shape within each female caste. *R* ^2^ represents the proportion of total Procrustes sum of squares (SS) explained by each term. *F*‐ and *Z*‐statistics, degrees of freedom (df), and associated *p*‐values (*p*) were obtained using 1000 permutations, three asterisks (***) indicate a *p*‐value of less than 0.001.

### 2.5 Allometric Analyses

To investigate whether wing shape changes systematically with body size within colonies, we fit a multivariate Procrustes linear model of shape on log(centroid size) with a caste × size interaction term to test whether allometric trajectories differed among castes. We then conducted within‐colony allometric analyses by regressing allometric regression scores on log(centroid size) separately for each colony within each caste. Preliminary pooled analyses revealed that between‐colony variation in body size dominated the allometric signal, particularly in gynes (Figure S4), and within‐colony analyses were therefore used to assess individual‐level size‐shape relationships. Within‐colony slopes and their 95% confidence intervals were used to assess the consistency and direction of size‐shape relationships across colonies within each caste.

### 2.6 Quantitative Genetics Analyses

We investigated heritability of wing shape in gynes and workers. Because males in Hymenoptera are haploid and develop from unfertilized eggs, patriline assignment and heritability estimation are not applicable to males; all quantitative genetic analyses were therefore restricted to the diploid female castes. Wing variation was estimated using Bayesian mixed‐effects models in the *MCMCglmm* (v2.36) package. A relatedness matrix (K) was constructed to represent genetic similarity among patrilines within colonies, and PC1 scores were modeled separately for gynes and workers with patriline and colony as random effects. Principal component scores summarize multivariate wing shape along orthogonal axes in Procrustes shape space, so that PC1, PC2, etc. represent independent directions of phenotypic variation. For the heritability analysis, we therefore treated PC1 as a quantitative trait and estimated genetic variance components within this axis rather than testing for genetic correlations between different PCs. Accordingly, these univariate models quantify how much variance along this primary shape axis is attributable to patriline and colony. Posterior estimates of patriline, colony, and residual variance were used to compute narrow‐sense heritability for each caste as *h* ^*2*^ *= V*_*A*_/(*V*_*A*_ *+ V*_*C*_ *+ V*_*R*_), where *V*_*A*_ is the patriline (additive) variance implied by *K, V*_*C*_ is the colony variance, and *V*_*R*_ is the residual variance, and 95% credible intervals were obtained from the posterior samples.

We then investigated whether genetic variation at the patriline level produced a shared “family signature” in wing shape across worker and gyne castes. This question was addressed using two separate but complementary analyses based on principal component scores, which provide orthogonal axes summarizing multivariate wing shape. First, we used distance analyses that quantified the multivariate Euclidean distances between pairs of individuals' wing‐shape configurations in the PC space. This allowed us to directly compare how similar worker‐gyne pairs were in their overall multivariate wing shape depending on whether they were full or half‐sisters within colonies. For this distance analysis, wing shape for each individual was first summarized in a low‐dimensional space using the first four principal components (PC1–PC4) from a PCA of Procrustes‐aligned landmark coordinates. For every colony, all possible worker–gyne pairs were formed, and the Euclidean distance between their PC scores was calculated as a measure of how different their wing shapes were. Each pair was labeled as “full sisters across caste” if the worker and gyne shared the same patriline, or “half sisters across caste” if they came from different patrilines within the same colony. These distances were then analyzed with linear mixed‐effects models that treated the pairwise distance as the response, the full versus half label as a fixed effect, and colony as a random effect. This approach directly tests whether full‐sister worker–gyne pairs were more similar in overall multivariate wing shape than half‐sister pairs, while accounting for shared nest environment. This approach directly tests whether full‐sister worker–gyne pairs were more similar in overall multivariate wing shape than half‐sister pairs, while accounting for shared nest environment and patriline‐pair structure.

Our second analysis for investigating shared genetic effects on wing shape used a patriline‐level genetic variance–covariance model (G‐matrix). In this framework, principal component scores again served as orthogonal axes of wing‐shape variation, and we treated each score as a quantitative trait. This analysis allowed us to quantify how patriline‐level genetic effects on specific wing‐shape axes (specifically PC1 and PC2) were shared between workers and gynes by estimating genetic variances and correlations for those specific PCs in each caste. To do this, wing shape was again reduced to principal components, and PC1 and PC2 were treated as quantitative traits in a multivariate mixed model. Four traits were defined: worker PC1, worker PC2, gyne PC1, and gyne PC2. A Bayesian mixed‐effects model was then fitted with patriline and colony as random effects, allowing the model to estimate how much patrilines contribute to variance in each trait and to covariances among traits. The resulting patriline variance–covariance matrix (the G‐matrix) described how patriline effects on these four orthogonal shape axes co‐vary, and from this matrix we derived genetic correlations that quantified how tightly the same patriline tended to move worker and gyne wing shapes together along each axis. This step evaluated how strongly matching shape axes were genetically coupled across castes, which directly informed the potential for independent caste‐specific evolution. By connecting cross‐caste resemblance in wing shape to an explicit quantitative genetic structure, this analysis allowed us to assess whether there was strong shared genetic control of particular shape axes across castes, rather than merely demonstrating that full sisters were more similar than half sisters.

## 3 Results

### 3.1 Wing Shape Variation Among Castes

We first investigated whether wing shape differed among the three *V. maculifrons* castes. We found that wing shape varied strongly among castes, with clear separation among gynes, workers, and males in the morphospace (Figure 2, Figure S1). The first two principal components explained a substantial proportion of the total shape variation (Figure 2A), with PC1 accounting for 29.16% and PC2 accounting for 18.47% of the variation across the full dataset. Wing shape differed significantly among castes (*p* = 0.001, Figure 2B–G, Table 1), with caste explaining 31.08% of the total shape variation (Table 1).

### 3.2 Allometric Scaling

A multivariate Procrustes linear model revealed a significant caste × size interaction (*p* = 0.001), indicating that allometric trajectories are not shared across castes in full Procrustes shape space. We conducted within‐colony allometric analyses by regressing allometric scores on log(centroid size) separately for each colony within each caste (Figure 3).

**FIGURE 3:**
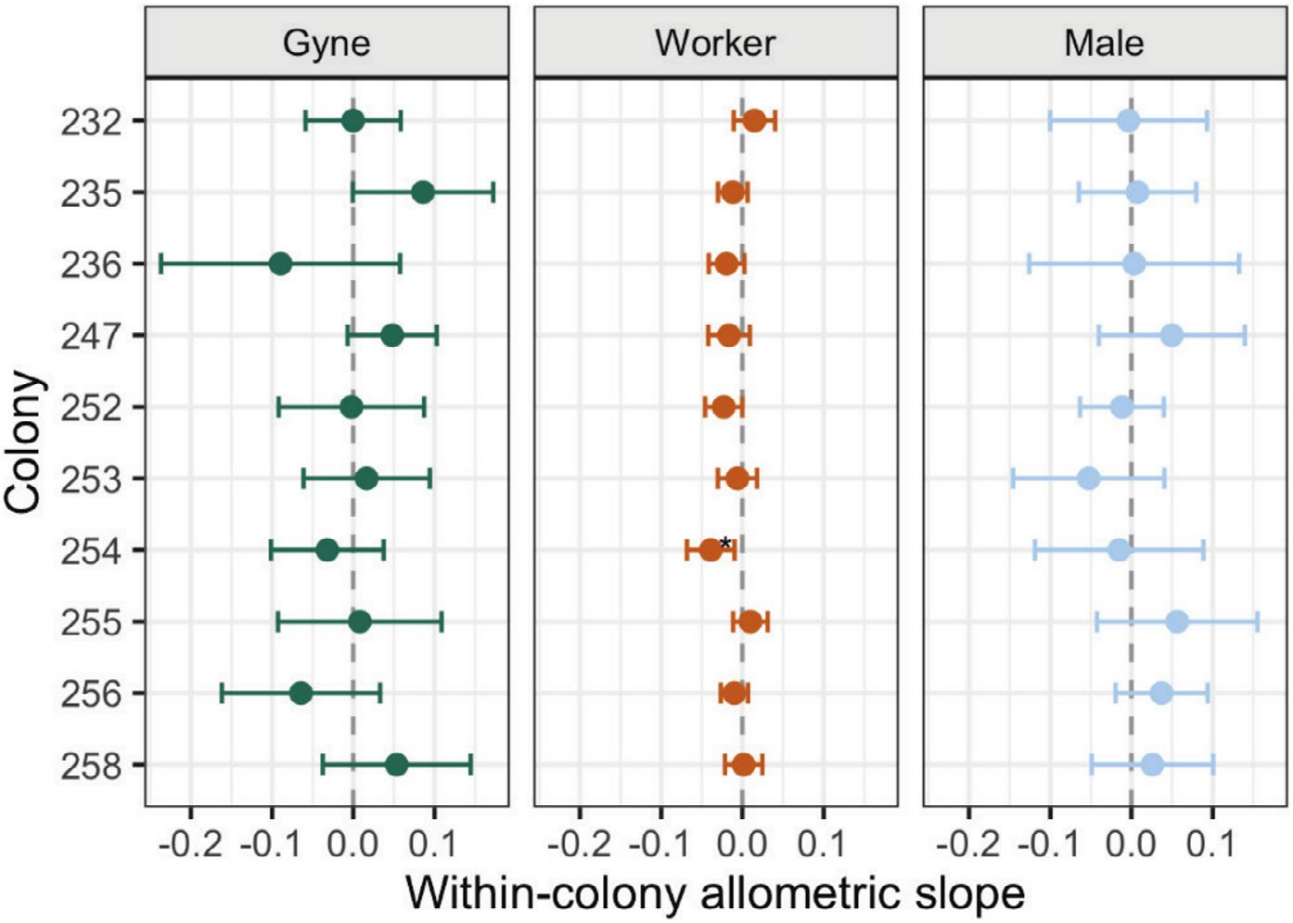
Within‐colony allometric slopes for wing shape by caste. Points show the slope of the regression of allometric score (RegScore) on log (centroid size) estimated separately for each colony within each caste. Error bars represent 95% confidence intervals. The dashed vertical line indicates a slope of zero (no allometry). Gynes showed no significant within‐colony size‐shape relationship in any colony, with slopes varying in direction across colonies (*n* = 275). Workers showed predominantly negative or near‐zero slopes, with one colony showing significant negative allometry (colony 254, *p* = 0.012, *n* = 599). Males showed no significant within‐colony allometry in any colony (*n* = 91).

Gynes showed no consistent within‐colony allometry, with slopes varying in direction across colonies and no colony reaching significance (*p* > 0.05 for all ten colonies). Colony 235 was notable as an outlier, with gynes substantially smaller in body size than those from other colonies and a positive but nonsignificant within‐colony slope (*p* = 0.070, Figure S4). The absence of consistent within‐colony allometry in gynes suggests that colony‐level body size variation, rather than individual developmental integration of size and shape, drives any apparent size‐shape relationship in this caste.

Workers showed predominantly negative or near‐zero within‐colony slopes, with one colony reaching statistical significance (colony 254, *p* = 0.012) and several others showing negative trends that did not reach significance. Although the directional tendency is suggestive, the weak and inconsistent signal across colonies does not support a strong claim of systematic negative allometry in workers.

Males showed no significant within‐colony allometry in any colony, with slopes near zero and wide confidence intervals reflecting the small sample sizes available per colony (*n* = 10). Absence of significance in males should therefore be interpreted with caution given limited statistical power at the within‐colony level. Together, these results indicated that size‐related developmental programs did not strongly couple wing shape to body size within colonies across any caste, and that the directional tendency in workers, while suggestive, was weak.

### 3.3 Colony‐Level Shape Variation

We next examined whether wing shape varied among colonies. We uncovered significant differences in wing shape between colonies nested within caste (*p* = 0.001, Table 1), explaining 19.15% of the total shape variation (Table 1). This indicates a substantial colony‐level component to wing shape variation, which could arise from both environmental and genetic influences. Regardless, even among individuals of the same caste, wing shape retained a clear colony‐specific signature.

### 3.4 Patriline‐Level Shape Variation and Heritabilities

Having established that caste and colony identity explain a large share of wing shape variation, we next asked whether workers and gynes belonging to different patrilines also differed in wing shape within colonies. To test for genetic contributions to wing shape, we examined the patriline effect on shape for each female caste. We found that patriline had significant effects on wing shape in both gynes and workers (*p* < 0.001, Table 1). In gynes, patriline explained 22.20% of the total variation (Table 1), whereas in workers, it explained 17.45% (Table 1). These effects were detected after accounting for colony differences. Therefore, these results are consistent with patriline‐associated genetic contributions to within‐colony wing shape variation.

We next examined the PCA scores of patrilines in order to further examine differences in wing shape between patrilines within colonies. Visualization of PCA scores by patriline further supports differences among patrilines in both castes. Individuals tended to separate by patriline within colonies (Figure 4). Although patriline clusters are not completely discrete, their partial separation suggests meaningful genetic effects on wing morphology.

**FIGURE 4:**
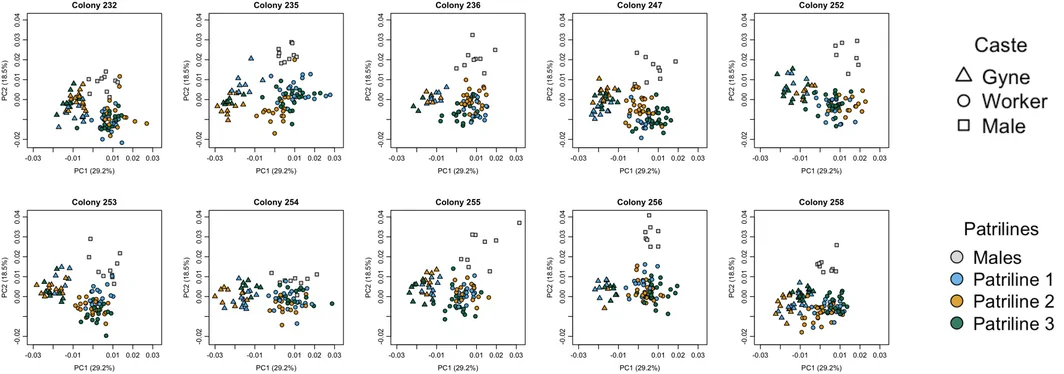
Principal component analysis of wing shape for members of different castes and patrilines for each of 10 colonies. Points show individual wasps in PC1–PC2 wing‐shape space, with symbol shape indicating caste and color indicating patrilineal identity. Within colonies, individuals from the same patriline tend to cluster in PC space, revealing consistent but partially overlapping patriline‐level differences in wing shape that contribute to within‐colony genetic variation.

We next estimated the narrow‐sense heritability of wing shape for each female caste (Figure 5A). We found significantly nonzero heritability values in wing shape for both gyne and worker castes. In workers, heritability was estimated at 0.275 (95% HPD: 0.091, 0.489) and in gynes, it was estimated at 0.264 (95% HPD: 0.103, 0.494). These results confirm that a substantial proportion of within‐colony variation is attributable to additive genetic variance.

**FIGURE 5:**
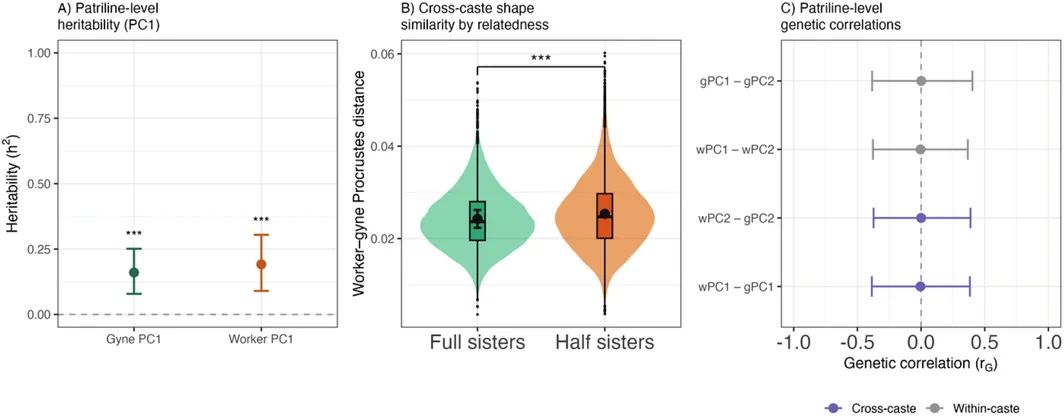
Genetic effects on wing shape in *V. maculifrons* . (A) Patriline‐level heritability estimates (*h* ^*2*^) for PC1 in workers and gynes. Points show posterior means and error bars show 95% highest posterior density intervals. Both worker and gyne PC1 showed significantly nonzero heritability (***95% HPD intervals exclude zero), confirming that patriline identity explains a substantial proportion of within‐colony variation in wing shape along the primary shape axis. (B) Empirical distributions of worker–gyne PC‐based Euclidean distances by relatedness. Violin plots and box plots summarize observed distances for full‐sister and half‐sister worker–gyne pairs. Black points and error bars show model‐predicted means ±1.96 SE from a linear mixed‐effects model with colony as a random effect. Full‐sister pairs were significantly more similar in wing shape than half‐sister pairs (***p < 0.0001, *t* = 10.22, df = 16,390). (C) Patriline‐level genetic correlations (*r*_*G*_) among wing shape principal components estimated from the multivariate MCMCglmm G‐matrix model. Points show posterior mean *r*_*G*_ and horizontal bars show 95% highest posterior density intervals. Cross‐caste correlations (purple) represent genetic coupling between matching shape axes in workers and gynes; within‐caste correlations (gray) represent genetic covariance among shape axes within each caste. All intervals overlap zero, indicating weak and imprecisely estimated genetic coupling across castes.

### 3.5 Genetic Correlation Modeling

We next asked whether patriline‐level genetic effects are expressed similarly in workers and gynes by quantifying cross‐caste genetic correlations in wing shape components. In other words, we tested whether workers and gynes from the same patriline tended to have more similar wing shapes than workers and gynes from different patrilines within colonies. To evaluate how genetic relatedness and patriline structure shape cross‐caste similarity in wing shape, we (1) modeled worker–gyne shape distances and (2) estimated patriline‐level genetic (co)variance in principal components of wing shape (Figure 5B,C).

Distance‐based mixed models confirmed that worker–gyne pairs that were half‐sisters were consistently farther apart in shape space than pairs of full‐sisters, indicating that highly related workers and gynes within colonies were more similar morphologically. In a model with colony as a random effect, half‐sister pairs were on average 0.00109 units farther apart in PC shape space than full‐sister pairs (estimate ± SE = 0.00109 ± 0.00011, *t* = 10.22, df = 16,390, *p* < 0.0001; *N* = 16,403 pairs, 10 colonies). Colony identity explained approximately 19% of the total variance in worker–gyne distance, with the remaining 81% at the individual pair level, indicating that colony identity contributes substantially to cross‐caste shape similarity but that a detectable patriline‐level family signature persists. In contrast, PC‐wise mixed models of ΔPC_1_–ΔPC_4_ show small estimates and ∣*t*∣ < 2 for the relationship effect on each individual axis, indicating that no single PC dominates the resemblance pattern. Instead, the full‐sister similarity arises from many small, diffuse differences across multiple PCs that only become clearly detectable when aggregated into a multivariate distance.

Multivariate quantitative genetic (G‐matrix) models at the patriline level further showed that, although there was substantial patriline‐level variance in each wing‐shape PC within both castes, genetic correlations across castes and components were weak. Patriline‐level correlations (*r*_*G*_) between matching PCs in workers and gynes were near zero (Figure 5C, worker PC1–gyne PC1: *r*_*G*_ = −0.0010, 95% HPD [highest posterior density] −0.37 to 0.37; worker PC2–gyne PC2: *r*_*G*_ = 0.0001, 95% HPD −0.39 to 0.37), and all cross‐PC, cross‐caste correlations similarly had wide HPD intervals overlapping zero. These near‐zero estimates with wide HPD intervals indicate that cross‐caste genetic coupling along specific PC axes was weak and imprecisely estimated. Overall, our analyses indicate that full‐sister workers and gynes (same patriline) have more similar wing shapes than half‐sister workers and gynes (different patrilines). Yet the patriline‐level genetic correlations between their wing‐shape components were weak, with wide HPD intervals overlapping zero. These findings imply that strong divergence in wing shape between workers and gynes can arise even when cross‐caste genetic correlations in wing shape are limited.

## 4 Discussion

Understanding how complex phenotypes evolve under shared genetic control is a central problem in evolutionary biology (Lande 1980; Pennell and Morrow 2013). In social insects, this problem is especially acute because queens, workers, and males share much of their genome yet experience strikingly different selective regimes (Wilson 1971; Bourke 2023). In this study, we integrated geometric morphometrics with detailed patriline information in the polyandrous social Eastern yellowjacket wasp (*Vespula maculifrons*) in order to better understand the environmental and genetic factors affecting wing shape. We show that wing morphology reflects both strong caste‐specific differences and substantial heritable variation, while remaining only weakly constrained by shared genetic architecture across castes. Together, our results demonstrate that wing shape in this social wasp system is influenced by a balance between genetic diversity generated by polyandry, colony developmental processes, environmental factors, and caste‐specific selective pressures associated with the division of labor.

### 4.1 Caste‐Specific Divergence in Wing Morphology

Wing shape differed strongly among gynes (prereproductive queens), workers, and males. Caste explained approximately one‐third of total shape variation and all pairwise caste comparisons showed significant divergence (Figure 2). This level of morphological differentiation is striking given that all castes retain wings and share most of their developmental genetic toolkit. Similar caste‐specific divergence in wing morphology has been reported in other social Hymenoptera (Perrard et al. 2012; Bourguignon et al. 2016; Peeters et al. 2020), suggesting that flight‐related traits are frequent targets of caste‐specific selection. The observed caste‐specific wing configurations are consistent with divergent flight demands associated with distinct life histories. Workers rely heavily on sustained, maneuverable flight for foraging and nest defense, whereas gynes primarily use flight for dispersal and colony founding, and males fly mainly during mating periods (Spradbery 1973; Ross and Matthews 1991; Hunt and Toth 2017). These functional differences likely impose contrasting selective pressures on wing shape, favoring distinct aerodynamic solutions despite a shared genome.

Morphological disparity patterns point to distinct selective regimes among castes. Male wings exhibited substantially greater shape variability than those of gynes or workers, a pattern that is consistent with either weaker stabilizing selection or a greater opportunity for sexual selection on male morphology (Baer 2003; Boomsma et al. 2005). In contrast, worker wings were tightly clustered in morphospace, suggesting strong stabilizing selection on a functionally efficient wing design optimized for frequent foraging flights. Together, these results reinforce the idea that caste specialization in social insects extends beyond behavior and body size to include fine‐scale aspects of flight morphology (Sumner et al. 2018; Friedman et al. 2019; Bergmann and Tonelli‐Sippel 2024; Orr, Hedrick, Murray, Pasupuleti, Kovacs, and Goodisman 2024).

### 4.2 Caste‐Specific Allometry in Wing Shape

Within‐colony allometric analyses revealed that the relationship between wing shape and body size differs among castes. However, allometric scaling of wing shape was weak or absent in all castes, and we concluded that developmental trajectories do not strongly constrain wing morphology (Figure 3).

Gynes showed no consistent within‐colony allometry, with slopes varying in direction across colonies and no colony showing significant allometric relationships. This absence of within‐colony size‐shape integration stands in contrast to what would be expected if gyne wing morphology were governed by a canalized developmental program tightly linking body size to wing shape. Instead, colony‐level size variation in gynes likely reflects environmental or nutritional differences among nests, but could also include colony‐level genetic and maternal effects (Smith et al. 2008; Kovacs, Hoffman, Marriner, and Goodisman 2010), and wing shape appears to vary independently of these colony‐level size differences at the individual level. The decoupling of wing shape from body size within colonies suggests that gyne wing morphology is not developmentally constrained by size in the way that might be expected for a caste that relies on sustained long‐distance flight for dispersal and colony founding.

Workers showed predominantly negative or near‐zero within‐colony slopes, with one colony reaching statistical significance (colony 254, *p* = 0.012) and several others showing negative trends that did not reach significance. Although the directional tendency is suggestive, the weak and inconsistent signal across colonies does not support a strong claim of systematic negative allometry in workers. Within colonies, larger workers may tend toward slightly different wing configurations than smaller workers, a pattern that could reflect size‐related task specialization. In many social Hymenoptera, worker body size is associated with division of labor (Oster and Wilson 1978; Wills et al. 2018; de Pletincx et al. 2021), with larger workers taking on different foraging or defensive roles that may impose distinct aerodynamic demands (Sumner et al. 2018; Friedman et al. 2019). Males showed no within‐colony allometry in any colony, consistent with relaxed stabilizing selection on male wing morphology and the limited flight demands males experience relative to workers (Linksvayer 2025), though the small per‐colony sample sizes for males limit the power of within‐colony analyses.

Together, these results indicate that size‐related developmental programs do not strongly couple wing shape to body size within colonies of *V. maculifrons* across any caste, and that the weak directional tendency in workers is unlikely to impose strong constraints on caste divergence. The independence of wing shape from body size within colonies leaves wing morphology free to evolve in response to caste‐specific selective pressures. This developmental modularity together with the quantitative genetic findings provides a coherent explanation for how strong caste divergence in wing morphology can arise and be maintained despite a shared genome.

### 4.3 Colony‐Level Variaion in Wing Shape

We found that colony identity explained a significant proportion of wing shape variation. This indicates a strong colony‐level signature in wing morphology. Differences in wing shape of individuals among colonies could arise from several nonindependent sources of variation. Individuals within a colony share aspects of their rearing environment, including nutrition, brood care, nest microclimate, colony demography, etc. (Smith et al. 2008; Kovacs, Hoffman, Marriner, and Goodisman 2010; Kovacs, Hoffman, Marriner, Rekau, and Goodisman 2010; Orr, Hedrick, Murray, Pasupuleti, Kovacs, and Goodisman 2024). At the same time, colonies differ in maternal genotype, the genetic composition of their patrilines, and indirect genetic effects mediated through social interactions, as well as other factors (Schwander et al. 2010). The significant patriline effects observed within colonies indicate that genetic variation contributes to wing‐shape variation among female offspring developing in the same colony. Therefore, among‐colony differences in wing shape should not be interpreted as evidence for a solely environmental origin of wing‐shape variation. Instead, they are consistent with combined contributions from shared developmental environment, colony genetic background, and socially mediated developmental processes. Future studies combining genome‐wide genotyping with direct environmental measurements, or experimental designs that decouple genotype and rearing environment, could estimate the relative contributions of these mechanisms.

### 4.4 Heritable Variation in Wing Shape

Within colonies, patriline identity explained a substantial fraction of wing shape variation in both workers and gynes. Patriline differences accounted for roughly 20%–36% of within‐colony variance. This provides strong evidence that wing shape is heritable within each female caste and that genetic differences among a queen's mates translate into measurable morphological differences among offspring. Notably, a prior study in the congener *V. flavopilosa* also detected a significant heritable component to wing venation shape (Perrard and Loope 2015). Similar patriline effects have been reported for other morphological and behavioral traits in social insects (Crozier and Page 1985; Baer and Schmid‐Hempel 2001; Tarpy 2003; Sankovitz et al. 2023), reinforcing the view that polyandry maintains variation within colonies and that “family signatures” in morphology are a common consequence of multiple mating.

Our finding of patriline associated wing‐shape variation is consistent with the view that social‐insect phenotypes arise through interactions between the effects of segregating genetic variation and environmentally responsive development. In honey bees and other social insects, caste‐associated morphology is strongly influenced by nutrition, brood care, and other social developmental factors. Transcriptomic and epigenomic studies have also shown that caste‐specific developmental trajectories are associated with distinct gene‐regulatory programs (Vojvodic et al. 2015; Wojciechowski et al. 2018). Strong environmental and social regulation of caste development does not preclude genetic contributions to morphological variation within a caste. Because *V. maculifrons* queens mate with multiple males, female offspring from different patrilines carry different paternal genomes while developing within the same colony. Therefore, a patriline‐associated contribution to within‐colony wing‐shape variation is possible. Our results extend previous analyses of size‐related morphological traits in these same individuals and colonies, which detected modest patriline effects for a subset of traits (Orr, Hedrick, Murray, Pasupuleti, and Goodisman 2024; Orr, Hedrick, Murray, Pasupuleti, Kovacs, and Goodisman 2024), and complement evidence for patriline‐associated forewing size and shape in the congeneric yellowjacket *Vespula flavopilosa* (Perrard and Loope 2015). However, it is important to note that our analyses do not identify loci causing wing shape variation or their regulatory mechanisms or permit a direct test of genotype‐by‐environment interactions underlying wing‐shape variation. Polyandry typically increases genetic diversity within a colony, which in turn increases the variability of wing phenotypes, potentially enhancing colony‐level performance and resilience (Crozier and Fjerdingstad 2001; Delaplane et al. 2015; Baer 2016). In this context, heritable variation in wing shape may contribute to functional diversity among workers or increase the likelihood that some offspring possess wing morphologies well suited to prevailing environmental conditions. Thus, polyandry not only increases genetic diversity per se but also preserves heritable variation in traits closely tied to fitness, including complex morphological traits such as wing shape.

### 4.5 Genetic Constraint Versus Caste‐Specific Evolution

A central goal of this study was to evaluate whether shared genetic architecture constrains the independent evolution of wing shape in workers and gynes. Our results show that patrilines generate detectable cross‐caste resemblance. Thus, there does appear to be a genetic correlation in wing shape between gynes and workers. However, we find that this correlation is relatively weak and only along specific shape axes. Distance‐based analyses revealed a detectable “family signature” across castes: worker–gyne pairs from the same patriline were more similar in overall multivariate wing shape than half‐sister pairs. Although the absolute difference is small and corresponds to only about 2% of the within‐colony range of pairwise distances, the large *t*‐value and very small standard error show that it is estimated with very high precision and represents a consistent, colony‐wide shift in multivariate wing shape. This indicates that patrilines exert consistent effects on wing morphology that span caste boundaries and confirms that genetic relatedness leaves a measurable imprint on cross‐caste phenotypic resemblance, echoing previous demonstrations of patriline‐level genetic influences on wing shape in social wasps (Perrard and Loope 2015).

However, when this resemblance was decomposed into specific shape components using quantitative genetic models, patriline‐level genetic correlations between worker and gyne wing shape PCs were weak and nonsignificant, with estimates centered near zero and wide intervals overlapping zero. In these models, PCs are treated as orthogonal axes of shape variation, so low and imprecise cross‐caste correlations along individual PCs indicate that genetic effects on worker and gyne wing shape are largely decoupled along the major axes of variation. No single principal component dominated the cross‐caste resemblance, and the family signature instead arose from many small, diffuse differences across multiple PCs. Together, these results suggest that while patrilines influence wing morphology in both workers and gynes, the specific ways in which genetic variation is expressed differ between the castes, and shared genetic variation does not rigidly constrain caste‐specific wing evolution along the primary shape axes.

This pattern is consistent with theoretical expectations for traits subject to divergent selection across phenotypic classes. Genetic correlations can limit independent evolution when selection pulls traits in opposing directions, but weak correlations allow phenotypes to approach caste‐specific optima despite a shared genome. More broadly, sociogenomic and quantitative‐genetic studies in social insects suggest that genetic architecture can become modular, with partially decoupled genetic effects across castes or trait modules, thereby facilitating the evolution of caste‐specific morphologies (Smith et al. 2008; Weitekamp et al. 2017). Our findings therefore support the view that genetic architecture in social insects can be sufficiently modular to permit caste divergence in complex traits such as wing shape (Schwander et al. 2010).

Together, these results indicate that patrilines contribute meaningfully to variation in each caste's wing shape, but that genetic effects on the main worker and gyne shape axes are only weakly coupled. As a consequence, wing‐shape divergence among castes in *V. maculifrons* can evolve largely independently despite only limited cross‐caste genetic correlations, in line with the idea that modular genetic architecture relaxes evolutionary constraints on caste‐specific trait evolution (Melo and Marroig 2015; Orr, Hedrick, Murray, Pasupuleti, Kovacs, and Goodisman 2024).

## Author Contributions

**Kayla A. Murray:** data curation (lead), formal analysis (equal), investigation (equal), writing – original draft (supporting), writing – review and editing (supporting). **Sarah E. Orr:** conceptualization (equal), data curation (equal), formal analysis (equal), funding acquisition (supporting), investigation (equal), methodology (lead), project administration (equal), supervision (lead), writing – original draft (lead), writing – review and editing (lead). **Michael A. D. Goodisman:** conceptualization (equal), formal analysis (lead), supervision (lead), writing – original draft (supporting), writing – review and editing (lead). **Emma L. Gosse:** data curation (supporting), methodology (supporting). **Rachel Hill:** data curation (supporting), methodology (supporting). **Jennifer L. Kovacs:** conceptualization (lead), data curation (equal), formal analysis (lead), funding acquisition (supporting), investigation (lead), methodology (lead), project administration (equal), software (lead), supervision (equal), visualization (equal), writing – original draft (supporting), writing – review and editing (equal).

## Funding

This work was supported by the Division of Integrative Organismal Systems (2019799), Division of Environmental Biology (2105033), and Agricultural Research Service (2023‐67012‐45432).

## Conflicts of Interest

The authors declare no conflicts of interest.

## Supporting information


**Table S1:** Distribution of data set revealing a total of 10 colonies, 275 gynes, 599 workers, and 91 males.
**Figure S1:** PCA of wing shape across colonies for all castes (A), workers (B), gynes (C), and males (D). Each color represents a unique colony.
**Figure S2:** Deformation grids by colony, separated by caste, using the overall mean caste shape as the reference (×30).
**Figure S3:** Deformation grids by patriline within colony, separated by caste and patriline, using mean colony caste shape as the reference (×60).
**Figure S4:** Pooled allometric trajectories for wing shape by caste. Regression scores summarizing the common size–shape allometric axis are plotted against log centroid size for gynes (green), workers (orange), and males (blue). Lines show fitted caste‐specific regressions from a pooled multivariate model. The apparent positive allometry in gynes reflects between‐colony body size variation rather than individual‐level size‐shape covariation; colony 235 gynes are substantially smaller than those from other colonies and drive the pooled gyne slope. Within‐colony analyses presented in Figure 3 provide a more appropriate assessment of individual‐level allometry.

## Data Availability

Wing images, landmark coordinates, morphometric data, and analysis code are available at GitHub repository https://github.com/jenniferkovacs/vespula‐wingshape‐caste‐genetics and at Dryad repository 10.5061/dryad.37pvmcw26. Microsatellite genotype data and inferred patriline assignments are available in Dryad at https://doi.org/10.5061/dryad.w6m905qv8.
