# Supplementary material for: Same Genes, Different Wings: Wing Shape Divergence Among Castes in a Social Wasp
Source: Ecol Evol. 2026 Oct 18;16(10):e74478. doi: 10.1002/ece3.74478 (PMC13678422; doi:10.1002/ece3.74478)
Supplement: Supplementary file 1 [file ECE3-16-e74478-s001.docx]

Same genes, different wings: wing shape divergence among castes in a social wasp

**Table S1.** Distribution of data set revealing a total of 10 colonies, 275 gynes, 599 workers, and 91 males.

| **Colony** | **Gynes** | **Workers** | **Males** | **Totals** |
| --- | --- | --- | --- | --- |
| **232** | 36 | 56 | 10 | **102** |
| **235** | 7 | 63 | 10 | **100** |
| **236** | 16 | 59 | 9 | **84** |
| **247** | 33 | 67 | 9 | **109** |
| **252** | 23 | 55 | 7 | **85** |
| **253** | 27 | 60 | 10 | **97** |
| **254** | 28 | 51 | 10 | **89** |
| **255** | 28 | 54 | 9 | **91** |
| **256** | 17 | 66 | 8 | **91** |
| **258** | 40 | 68 | 9 | **117** |
| **Totals** | **275** | **599** | **91** | **965** |

**Figure S1**. PCA of wing shape across colonies for all castes (A), workers (B), gynes (C), and males (D). Each color represents a unique colony.

 

**Figure S2**, deformation grids by colony, separated by caste, using the overall mean caste shape as the reference (x30)

**Figure S3**, deformation grids by patriline within colony, separated by caste and patriline, using mean colony caste shape as the reference (x60)

**Figure S4.** Pooled allometric trajectories for wing shape by caste. Regression scores summarizing the common size–shape allometric axis are plotted against log centroid size for gynes (green), workers (orange), and males (blue). Lines show fitted caste-specific regressions from a pooled multivariate model. The apparent positive allometry in gynes reflects between-colony body size variation rather than individual-level size-shape covariation; colony 235 gynes are substantially smaller than those from other colonies and drive the pooled gyne slope. Within-colony analyses presented in Figure 3 provide a more appropriate assessment of individual-level allometry.
